# Relationship Status and Sexual Behaviors in Post-Pelvic Inflammatory Disease (PID) Affected Urban Young Women: A Sub-Study of a Randomized Controlled Trial

**DOI:** 10.23937/2469-5823/1510088

**Published:** 2018-01-10

**Authors:** Lisa Tabacco, Shang-en Chung, Jamie Perin, Steven Huettner, Arlene Butz, Maria Trent

**Affiliations:** 1 Division of General Pediatrics and Adolescent Medicine, School of Medicine, Johns Hopkins University, USA; 2 Armstrong Institute, Johns Hopkins University, USA; 3 Division of Global Disease Epidemiology and Control, Johns Hopkins Bloomberg School of Public Health, Johns Hopkins University, USA

**Keywords:** Adolescent, Pelvic inflammatory disease, Reproductive health, Sexual behavior

## Abstract

Adolescent and young adult women disproportionately experience Pelvic Inflammatory Disease (PID) as a complication of Sexually Transmitted Infections (STIs). This study seeks to understand the relationship context, changes in sexual behavior, and impact of partner sexual behavior on recurrent STI diagnoses at 3-months post-diagnosis. Adolescents and young adult women 13–25 were recruited from an outpatient disposition from an outpatient clinic, and pediatric and adult emergency rooms. Participants received treatment at baseline and follow-up at 2-weeks, 1-month and 3-month post-diagnosis, including interviews about personal and partner sexual behaviors and STI screening (n = 94). At the 2-week interview, 53% of participants (50/94) believed they could acquire an STI from their current partner if they did not use a condom. However, at 3-month follow-up only 35% reported condom usage at last sex. At 3-month follow-up, 55% (50/91) of participants were still in a sexual relationship with the previously reported partner and 38% of participants who reported they could get an STI from their partner were diagnosed with an STI; compared with 25% of participants who predicted that they could not get an STI (OR 1.85; 95% CI: 0.67–5.30). There was no association between maintaining the same partner and having an STI at 3-months (OR 0.5; 95% CI: 0.27–1.96). Most young women diagnosed with PID report exclusive relationships, but are simultaneously aware of their risk for recurrent STIs. Given the short-term stability of many relationships, couples interventions are an unexplored opportunity for prevention of recurrent STIs after PID.

## Introduction

Pelvic Inflammatory Disease (PID) is often caused by a complication of untreated Sexually Transmitted Infections (STIs). PID-diagnosed urban young women experience disproportionately higher rates of recurrent STIs [1]. The 2015 Sexually Transmitted Diseases Treatment Guidelines released by the Centers for Disease Control and Prevention (CDC) recommend that the sexual partners of patients with Pelvic Inflammatory Disease (PID) be treated regardless of the biological etiology for the affected female patient [2]. Given the difficulties associated with successful partner notification and treatment, use of public health strategies such as prescribing empirical partner treatment, often referred to as expedited partner therapy, are being implemented across the country. Unfortunately, giving partners medicine does not address the underlying relationship issues and behaviors that may continue to place the couple at risk for recurrent STIs and the index patient at risk for recurrent PID and associated sequelae such as tubal infertility and chronic pelvic pain [1].

Adolescents and Young Adults (AYAs) are at a higher risk for STIs because they engage in more concurrent partnerships, multiple sex partners, and unprotected sex than their adult counterparts [3–5]. Sexual partnerships that overlap in time, or concurrent partnerships, have been identified as effective transmission agents of STIs [6]. The acquisition of a new partner has also been identified as a predictor of incident STI infections [7,8]. Data suggest that for urban young women, exclusive, monogamous relationships may be hard to establish and transitioning to a new partner may not be protective against recurrent STIs [3]. In this analysis, we aim to describe the relationship context of AYAs with PID, the relationship changes that occur after a PID diagnosis, condom usage following PID diagnosis, and the perceived risk for subsequent STIs by PID-affected patients. We also examine longitudinal STI outcomes to evaluate the relationship between partner change and STI results 3-months after the PID diagnosis.

## Methods

We conducted a sub-study of 94 AYA women aged 13 to 25-years-old enrolled in the Technology Enhanced Community Health Nursing (TECH-N) study, a large randomized controlled trial of a community health nursing intervention with text messaging support designed to reduce recurrent STIs after PID. The TECH-N study has been previously described in the literature [9], but is briefly reviewed here. Patients with mild-moderate PID were recruited from outpatient clinics and pediatric and adult emergency departments of a large academic center situated in a community with high STI prevalence. Intervention participants (n = 47) were randomized via block design, received medication reminders, appointment reminders, and sexual health support text messages as well as a clinical assessment within 72-hours and STI/HIV prevention intervention (Sister-to-Sister Teen) [9,10] delivered via home visit by a community health nurse. All participants received standard of care per the CDC guidance, a complete course of antibiotics to treat PID, and a total of $60 for their participation in the baseline, 2-week, 1-month, and 3-month study visits, with follow-up success exceeding 90%. For this sub-study, additional relationship questions were added to the 2-week face-to-face outreach interview. These questions were meant to assess relationship status, relationship expectations, and ongoing relationship intentions, both in terms of maintaining the relationship and future condom use and negotiation after an STI diagnosis.

All participants provided baseline data on demographics (age, race/ethnicity, insurance status), relationship status, and sexual behaviors using an audio computerized assisted self-interview as well as biological specimens to evaluate for *Neisseria gonorrhoeae, Chlamydia trachomatis, Trichomonas vaginalis* and *Mycoplasma genitalium* at baseline, 1- and 3-months. Due to the sample size, a single category (positive STI) was created to signify this status in analysis. Condom use data at last sex was obtained at baseline, 1-month, and 3-months. A variable was generated for STI positivity at 3-months for the purpose of this study as reporting of the biological outcomes are a part of the main study. During the 2-week interview, participants were asked to describe the status of the relationship with their current sexual partner as main or casual. Main partners were defined as someone the participant had previously had sex with and had serious feelings for that partner a casual partner was defined as someone the participant had previously had sex with at least once or infrequently and did not have serious feelings for that partner [5]. Participants were also asked to predict their perceived risk for STIs if the sexual relationship continued, and their behavioral intentions if they discovered their sexual partner had concurrent sexual partners. Descriptive, bivariate, and logistic regression analyses were used to evaluate the relationship between partner change and STI at 3-months controlling for group assignment. The Institutional Review Board approved the randomized trial, including the relationship sub-study questions and written informed consent was obtained from participants.

## Results

**Figure 1:**
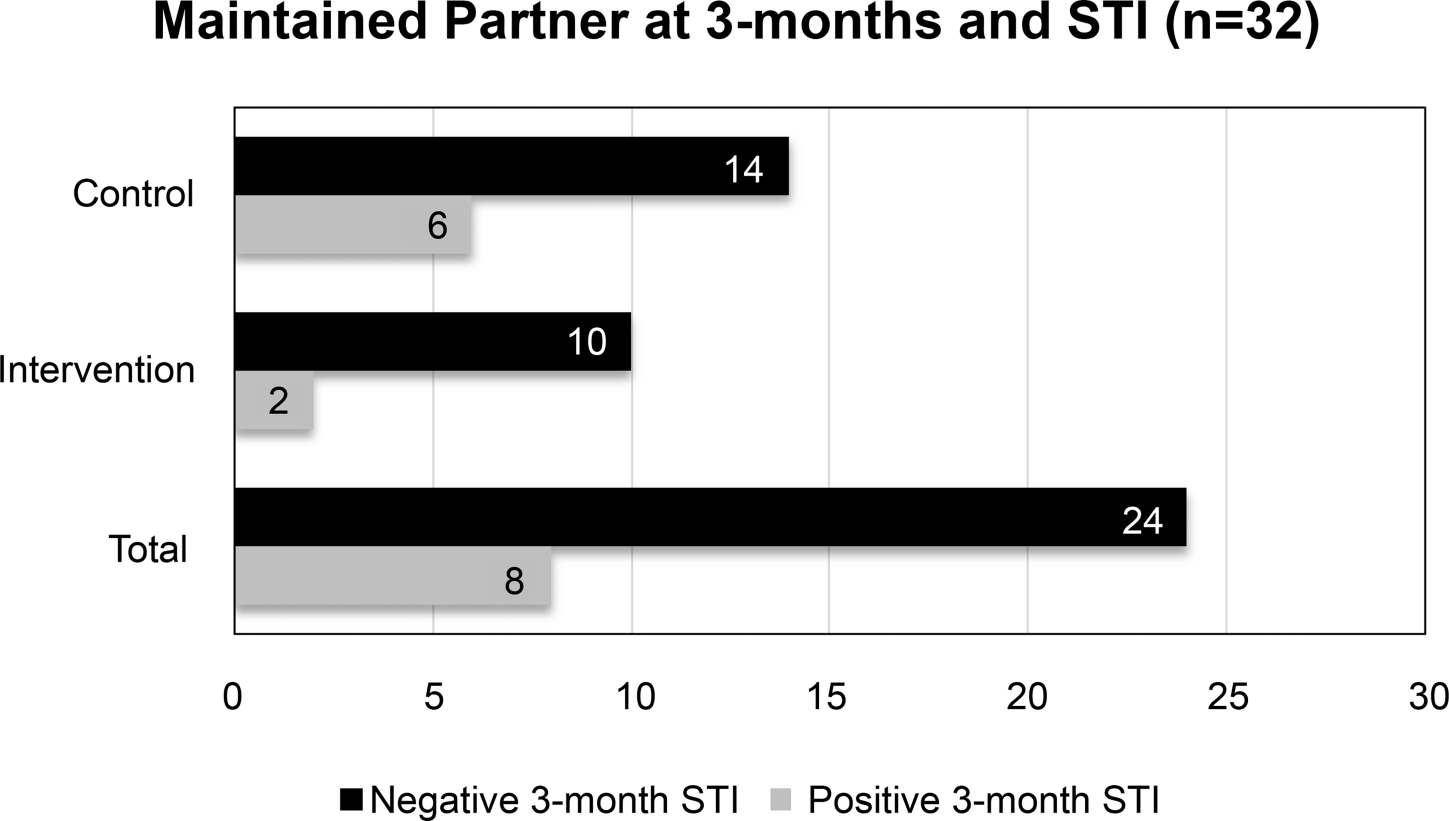
Relationship maintenance and STI outcomes at 3 months.

Of the 94 participants, 47 were in the control group, 47 in the intervention, and 3 were lost to follow-up. Most participants were low-income (77%), African American (93%) young women with a mean age of 18.5 (SD 2.2) who described being in exclusive monogamous relationships (84%) (Table 1). At baseline, participants reported an average of 1.3 (SD 0.9) sexual partners in the 3-months preceding their PID diagnosis, further suggesting a period of exclusivity. At the 2-week interview, 53% of participants (n = 50) believed they could acquire an STI from their current partner if they did not use a condom. Ninety-six percent (n = 90) of participants claimed they would alter their behavior if they found their partner to have another concurrent partner. When asked how they would alter their behavior, 70% said they would discontinue having sex with their partner and 18% would end the relationship. As seen in Table 2, at 1-month, 49% of respondents did not use a condom at last sex, compared with 32% who reported using a condom. At 3-months, 55% (50/91) of participants were still with the same sexual partner described in the baseline interview. However, although not statistically significant, intervention group participants were less likely to maintain the same sexual partner at 3-months (OR 0.80; 95% CI: 0.32–1.99). Thirty-eight percent of participants who reported they could still get an STI from their partner at the 2-week interview were diagnosed with an STI at 3-months; compared with 25% of participants who predicted that they could not get an STI (OR 1.85; 95% CI: 0.67–5.30), controlling for group assignment. Of participants who maintained their sexual partner, fewer intervention participants had a positive STI at 3-months as demonstrated in Figure 1. At 3-months, more participants (35%) reported condom use at last sex, with 27% reporting no condom use at last sex and a greater proportion of unknown condom use (38%). Although 28% of participants who maintained the same partner were diagnosed with an STI at 3-months, this was less than the 35% of participants who did not maintain the same partner. However, this association was not statistically significant (OR 0.93; 95% CI: 0.76–1.14), controlling for group assignment.

## Discussion

This study demonstrates that most PID-affected urban young women describe being in exclusive relationships with a main partner at the time of diagnosis. Despite reported relationship exclusivity, over half consider themselves at risk for future STIs at the 2-week interview. Although many participants assert that they would end their sexual relationships with a partner believed to be concurrent based on STI testing and/or presumptive diagnoses, about half did not. Work by Matson, et al. suggests that among a similar study population the average length of an adolescent romantic relationship is 16 months with a median of 7 months among their sample [4]. While this study examined the immediate 3-month window following PID diagnosis, this work suggests that there is stability within the adolescent relationships even in the sentinel event of a complicated STI (PID). Although the STI outcomes data did not reach statistical significance, the trend observed among stable partners is suggestive of a protective effect that could be leveraged for public health intervention with communal coping and subsequent sexual negotiation improvements in the aftermath of a PID diagnosis. The intervention group participants were less likely to maintain their sexual partner, however, that was not protective of STI acquisition. Possible explanations for the difference among intervention and control participant relationship stability could be the increased education and emphasis on safer sex practices following the PID diagnosis. The outcomes from this analysis demonstrate that there is short-term relationship stability in AYA couples affected by a PID diagnosis offering healthcare providers a window of opportunity for public health prevention.

The 3-month study period following a PID diagnosis for these AYA women represented a limited window, with an emphasis on the immediate aftermath of a serious STI complication. The 2-week treatment period for PID with subsequent 2-week interview were meant to capture the participants’ impressions of their diagnosis and the impact on their relationships at the moment when the sexual relationship could recommence following the completion of treatment. Our findings show that condom use did not approach desired levels, with only 32% (n = 30) of participants reporting condom use at 1-month and a minor increase to 35% (n = 33) at 3-months. These low levels of condom use represent a focus area for future healthcare interventions. The implied trust, or renegotiation of trust, involved in choosing not to use a condom following a PID diagnosis further represents an area of future research into trust and intimacy negotiations following the implication of concurrent partnerships.

The sample population discussed in this study represents mostly low-income African-American AYA women in an urban area with access to a large, academic hospital. A multi-site study looking at relationship stability and behaviors following an STI diagnosis would be valuable to understand relationship behaviors across demographics and location. It cannot be ignored that minority youth experience disproportional STI rates in the United States as well as the social and racial implications of positive STIs on the life course [2]. The continued risk-taking seen in our sample following PID diagnosis may have greater implications beyond relationship status than our survey was able to capture. This study shows that peer networks in communities with prevalent STI rates may be impacted by the news of a positive STI, and in this case, a PID diagnosis, less than anticipated. Further study is needed to understand coping strategies and future sexual health decisions within this population.

The findings from this work must be considered in light of several limitations. We have used a small sample of AYA women from a single trial, academic center, and community, indicating that the findings may not be generalizable to other groups. Our small sample consists of only trial participants who were asked these relationship follow-up questions. We assessed their relationship status over a limited 3-month period and there may be more variability over longer periods of study. Even so, these findings suggest that the 3-month period following an STI diagnosis is a critical time for intervention with young couples that wish to continue their relationship.

In summary, almost half of AYA women remain with their sexual partners after PID even though most participants perceive an STI diagnosis as a violation of monogamy and are not accepting of being in a concurrent relationship. Many participants in this study predicted STI recurrence if they did not use a condom with their current partner, but did not prevent re-infection, emphasizing ongoing risk-taking behavior in the face of perceived susceptibility. This suggests that girls may be ambivalent about relationships and/or practical about the realities of partner selection when residing in an urban community with high rates of disease [3–5], resulting in risk-laden decisions to maintain the relationship. Based on participants’ history of STI positivity and PID, condom use interventions remain relevant. However, our evidence suggests that condom usage slightly increases over time following PID diagnosis and treatment. Young couples may have sufficient short-term stability despite the interim STI diagnosis to attempt novel intervention strategies such as simultaneous couples STI treatment with sexual health counseling and condom negotiation training to decrease STI reinfection [4]. Further study is needed to confirm these preliminary findings and to evaluate the effectiveness of couples’ interventions following the acute PID diagnosis to decrease recurrent infections and associated longitudinal reproductive health sequelae.

## Figures and Tables

**Table 1:** Demographics of PID affected women (n = 94).

	%, n

Race	

Black/African American	93%, 87
	
White	1%, 1
	
Hispanic/Latino	3%, 3
	
Other	3%, 3

Age at enrollment (mean, sd)	18.5 (2.2)

Insurance Status	
Private	13%, 12
	
Medicaid	77%, 72
	
Self-Pay	11%, 10

Relationship Status at enrolment	
Main Partner	84%, 79
	
Casual Partner	16%, 15

Condom Use at enrolment	
Yes	15%, 14
	
No	17%, 16
	
Unknown	68%, 64

Number of lifetime partners (mean, sd)	5.6 (6.6)

Number of partners past 3 months (mean, sd)	1.3 (0.9)

%: Percentage; n: Number; sd: Standard Deviation.

**Table 2:** Relationship status, Condom Use, and STI prevalence of PID affected women (n = 94).

	Total %, n	Intervention %, n	Control %, n	p-value	OR	95% CI
Relationship Status at enrollment						
Main Partner	84%, 79	-	-	-		
Causal Partner	16%, 15	-	-	-		
Relationship Status at 3-months	-	-	-	0.74		
Maintained Sexual Partner	55%, 50	51%, 23	57%, 25	-	0.80	0.32 – 1.99
Different Sexual Partner	45%, 41	49%, 22	43%, 19	-		
2-week interview report of STI risk						
Yes	53%, 50					
No	47%, 44					
STI at 3-months	-	-	-	1.00		
Positive	31%, 28	31%, 14	32%, 14	-	0.93	0.76 – 1.14
Negative	69%, 61	69%, 31	68%, 30	-		
Condom Use at enrollment	-	-	-	-		
Yes	15%, 14	-	-	-		
No	17%, 16	-	-	-		
Unknown	68%, 64	-	-	-		
Condom Use at 1-month	-	-	-	0.71		
Yes	32%, 30	34%, 16	32%, 14	-		
No	49%, 46	45%, 21	52%, 23	-		
Unknown	19%, 18	21%, 10	16%, 7			
Condom Use 3-months				0.44		
Yes	35%, 33	33%, 15	36%, 16			
No	27%, 25	35%, 16	20%, 9			
Unknown	38%, 36	35%, 16	43%, 19			

%: Percentage; n: Number; CI: Confidence Interval; OR: Odds Ratio.
